# Seasonal trends of the polyp expansion and nutritional condition of *Alcyonium acaule* (Octocorallia, Alcyonacea)

**DOI:** 10.7717/peerj.12032

**Published:** 2021-10-14

**Authors:** Lucia Rizzo, Ida Fiorillo, Sergio Rossi

**Affiliations:** 1 Department of Integrative Marine Ecology, Stazione Zoologica Anton Dohrn, Napoli, Italy; 2 Institute of Marine Sciences (ICM-CSIC), Barcelona, Spain; 3 National Interuniversity Consortium for Marine Sciences (CoNISMa), Roma, Italy; 4 Department of Biological and Environmental Sciences and Technologies, University of Salento, Lecce, Italy

**Keywords:** Marine animal forest, Habitat-forming species, Anthozoans, Polyp expansion, Organic matter, Octocorals, Seasonal changes

## Abstract

The ecological physiology of anthozoans, as well as their resistance to stressors, are strongly influenced by environmental factors and the availability of resources. The energy budget of anthozoans can vary seasonally in order to find an equilibrium between the available resources and respiration, polyp activity, growth, and reproduction processes. The variation in the biochemical composition of the animal tissues in these organisms results from a combination of the productivity processes of the water column coupled with the reproductive effort and potential starvation periods of the anthozoans. Here, the seasonal variation in the polyp activity of a slow-growing passive suspension feeder, the octocoral *Alcyonium acaule*, as well as their carbohydrate, protein and lipid contents, was investigated in a warm temperate environment using *in-situ* observations and biochemical analyses. Polyp activity exhibited a significant variability that was moderately dependent on season, while an aestivation phenomenon in *A. acaule* (*i.e.*, a resting period in which the anthozoan is not capable of any polyp activity) during the warmer months is clearly observed. Carbohydrate concentrations in the coral species showed a significant increase in the late winter and spring seasons, and the lipid content increased during the spring. A higher abundance of lipids and carbohydrates coincided with a higher primary productivity in the water column, as well as with the octocoral reproduction period. In late autumn, there was a depletion of these biomolecules, with protein levels exhibiting great variability across sampling times. Complex alterations driven by climate change could affect the energy fluxes that depend on the dead or alive particles that are intercepted by marine animal forests. The obtained findings show a food shortage in late summer and autumn of the benthic suspension feeder *A. acaule* through the integrative descriptors of the ecophysiology of these anthozoans. This research contributes to the knowledge of energy storage capabilities in benthic suspension feeders in general, highlighting the importance of understanding the limits of resistance to starvation periods through these indicators.

## Introduction

Coastal benthic ecosystems represent a hotspot of marine biodiversity, forming different habitats and providing shelter to benthic and demersal biota (Bianchi & Morri, 2000; Rossi et al., 2019a; Rossi & Rizzo, 2020). Climate change, acting simultaneously with the growing frequency of anthropic activities, can affect habitat-forming species and alter coastal trophodynamics (Pusceddu et al., 2016; Rizzo et al., 2017, 2020). These human-driven stressors can influence energy fluxes related to the availability of food quantity and quality, and threaten the fitness of sensitive species (Rossi et al., 2019a; Rossi & Rizzo, 2020). Seasonal patterns in growth, reproduction, and abundance of marine benthic suspension feeders are well recognized, revealing the strong dependence of coastal ecosystems on large environmental variability (Clarke, 1988; Valiela, 1995). Understanding the relationship between the temporal changes of environmental factors, water column resources (*e.g.*, nutrients or food availability), and the response of benthic organisms, represents a crucial step in coupling physical and biological processes in the ocean.

Coma et al. (2000) reported a seasonal pattern in the activity in benthic suspension feeders driven by temperature in cold temperate seas, showing an autumnal decrease in activity and in secondary production, a minimum in the winter period, and an increase during spring and summer (Clarke, 1993). Antarctic gorgonians collected in the Eastern Weddell Sea and the Bransfield Strait during primary productivity depletion had a diet based on resuspended material, suggesting a consumption of phytoplankton detritus during the winter (Elias-Piera et al., 2013). However, in warm temperate seas, such as the Mediterranean basin, the responses of all benthic suspension feeders are largely heterogeneous (Boero et al., 1986; Boero & Fresi, 1986; Coma et al., 2000). During the summer season, some benthic species face an adverse feeding period and seem to be affected by aestivation (summer dormancy) instead of winter dormancy, including ascidians (Turon, 1992; Turon & Becerro, 1992), bivalves and polychaetes (Ramón, Abelló & Richardson, 1995; Arneri, Giannetti & Antolini, 1998; Sardá, Pinedo & Martin, 1999), and anthozoans (Coma et al., 1998; Garrabou, 1999). Generally, benthic species show the highest reproductive output and growth rate in late winter, spring and early summer (Turon, 1992; Turon & Becerro, 1992; Coma et al., 1998; Garrabou, 1999). Cold-water and warm-water species live in the Mediterranean Sea, showing a coexistence in the array of climatic and hydrologic conditions. The resulting high species richness of this sea is recognized as a hotspot for biodiversity (Bianchi & Morri, 2000).

Different ecological factors and related interactions affect seasonal cycles of benthic species (Gili & Hughes, 1995; Sardá, Pinedo & Martin, 1999). Temperature, water movement, and food availability have been identified as crucial environmental factors affecting the dynamics of benthic suspension feeders, such as in *Corallium rubrum* (Rossi, Rizzo & Duchêne, 2019), *Paramuricea clavata* (Coma et al., 1994), *Alcyonium acaule* and *Parazoanthus axinellae* (Garrabou, 1999). In the Mediterranean Sea, changes in temperature, primary productivity, and water stratification create different conditions in food availability. Benthic suspension feeders and their metabolic responses depend on the supply of food (Coma et al., 1998; Rossi et al., 2006) associated with the short-time-scale variability of near-bottom seston (Rossi & Gili, 2007; Rossi & Rizzo, 2021). In late winter/early spring, the stabilization of surface seawater influences changes in the supply of food, and in autumn, the thermocline breaking mixes the waters and related food (Estrada, 1996). During the summer, the strong stratification of the water column leads to a depletion of suspended materials. Polyp expansion (*i.e.*, the opening of polyps in anthozoans, Rossi, Rizzo & Duchêne, 2019) can be stimulated by increasing the frequency of food inputs (Tsounis et al., 2006; Rossi & Gili, 2007; Rossi & Rizzo, 2021). This triggers a response in passive suspension feeders in front of changes in seston availability, supporting their feeding activity (Coma et al., 1994; Garrabou, 1999; Rossi, Rizzo & Duchêne, 2019). However, while climate change and related problems of energy availability have been shown to affect the metabolic rates of these organisms, the underlying basic mechanisms regulating ecophysiological processes in warm, temperate benthic suspension feeders remain poorly investigated.

Soft corals (no skeletal axis) belong to the order Alcyonacea, a main structural group of benthic colonial invertebrates found across all latitudes and depths, from shallow waters to the deep sea (Dinesen, 1983; Lasker, Gottfried & Coffroth, 1983; Spalding et al., 2001; Mortensen & Buhl-Mortensen, 2004; Roberts et al., 2009). *Alcyonium acaule* Marion, 1878 (Cnidaria: Octocorallia: Alcyonacea) is colonial with a sessile soft coral thick-bodied and finger-like lobes. This species grows to form a cluster up to 20 cm in height. It is a slow growth organism with a life span of >20 years (Teixidó, Garrabou & Harmelin, 2011). This soft coral is abundant in the Mediterranean Sea, in the pre-coralligenous and coralligenous bioconcretions, *i.e.*, outcrops consist of coralline algal frameworks that grow in dim light conditions (Fiorillo et al., 2013; Ambroso et al., 2013; Martin et al., 2014). In certain cases, this species forms dense aggregations, which reinforces its role in community organization (Garrabou, 1999; Ambroso et al., 2013).

The aim of this study was to evaluate seasonal differences in polyp expansion and tissue concentrations of proteins, carbohydrates, and lipids of the benthic invertebrate *A. acaule* in a temperate sea (*i.e.*, Mediterranean Sea) in relation to temporal coupling with environmental variables. The variation in the biochemical composition of the tissue of the soft coral *A. acaule* may depend on productivity fluctuations of the water column, and seasonal fluctuations in seston quantity and quality (Rossi et al., 2003; Rossi & Gili, 2005).

## Materials & Methods

### Study area and sampling

**Figure 1:**
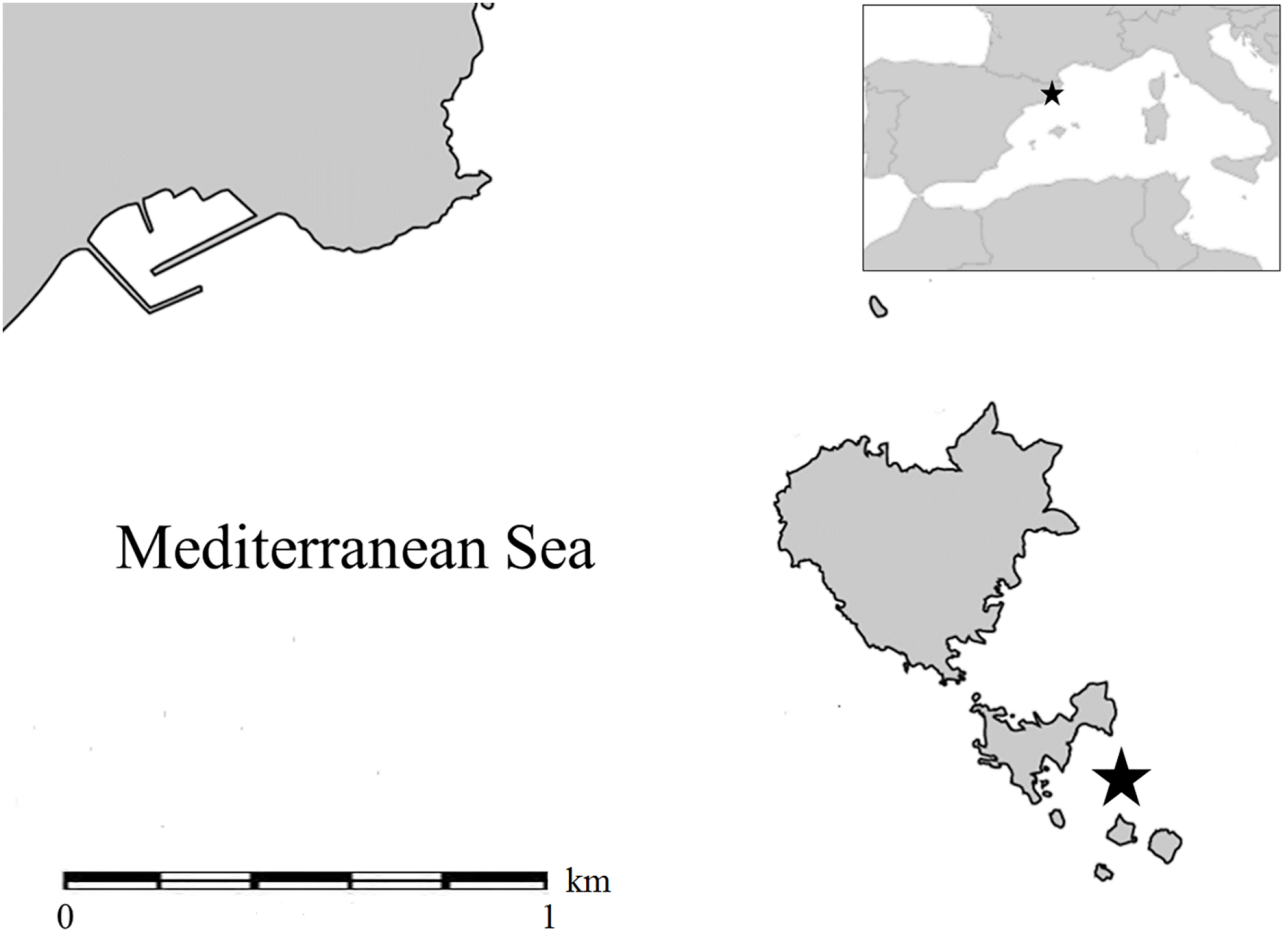
Map of the sampling location indicated with a star (Tascons Grossos, Marine Protected Area of the Medes Islands) in the Mediterranean Sea.

One hundred and twenty *A. acaule* colonies were observed and sampled in the Marine Protected Area of the Medes Islands (NW Mediterranean Sea, Catalonia, Spain), in Tascons Grossos (40°02′55″’N, 3°13′30″E, Fig. 1). Sampling was carried out at 11–19 m depth in a channel of rocky blocks by SCUBA diving from June 2002 to June 2003. Dense *Alcyonium acaule* colonies live on rocky bottom at 10–50 m depth in the study area, together with other benthic suspension feeders and coralline algae (Gili & Ros, 1985). The channel is characterized by alternating currents (north/south and south/north) that can reach high speeds (Rossi, 2002; Rossi & Gili, 2007; Rossi, Rizzo & Duchêne, 2019).

### Environmental variables

Sea water temperatures were recorded three times each month at two different depths (5 and 20 m depth), ~100 m away from the sampling area (Rossi & Gili, 2005), using an inverted thermometer according to Cebrián, Duarte & Pascual (1996). Water transparency and seston abundance were evaluated applying the Secchi disk protocol (Preisendorfer, 1986). A vertical Secchi disk (VSD) was observed each 10–12 days from a boat 20–30 m from the sampling point. Observations were always made between 10:00 a.m. and 12:00 p.m. to avoid interference from incident light (Preisendorfer, 1986).

### Seasonal polyp expansion

Polyp expansion was observed in *Alcyonium acaule* over the entire year. Colonies with expanded polyps or completely contracted polyps were observed visually and registered monthly three times a season (100 healthy colonies grouped in sets of 10 were observed each time randomly) by SCUBA diving. Only colonies showing polyps with 100% expansion were assigned as expanded polyps, while colonies with closed polyps were recorded as contracted polyps. Colonies in between these stages were not counted. This alcyonarian also exhibits dormant colonies that were also recorded (*i.e.*, those animals with contracted polyps and a thin film covering the surface, Garrabou, 1999).

### Biochemical analyses

During the sampling, apical branches (no more than one cm length) were cut and transported in a cooler (4 °C). Samples were frozen in liquid nitrogen within 30–40 min of sampling. The samples were held at −80 °C, freeze-dried at −110 °C and a pressure of 100 mbar, and then stored frozen at −20 °C pending biochemical analyses.

Results have been reported in μg Protein-Carbohydrate-Lipid mg^−1^ OM (Organic Matter). Organic matter was obtained by taking 10–15 mg of the tissue each month (over 1 year), heating the tissue at 80 °C for 48 h and weighing the sample to obtain dry weight. The same tissue was ashed for 4 h at 500 °C. The difference between dry weight and ash weight was considered organic matter (Rossi et al., 2006).

All analyses were performed spectrophotometrically (colorimetrically) following the protocol of Rossi et al. (2006). For proteins, a 10–15 mg piece of tissue was homogenized in 6 ml 1 N NaOH, and quantified using the method of Lowry et al. (1951), using bovine serum albumin as the standard (Sigma-Aldrich, St. Louis, MO, USA). For carbohydrates, a 15–20 mg piece of each branch was weighed in a microbalance (precision: ±0.01 mg), and the tissue was homogenized in 6 ml of double distilled water. Carbohydrates were quantified using the method reported by DuBois et al. (1956), using (D)-glucose as standard (Sigma-Aldrich, St. Louis, MO, USA). For lipids, a 15–20 mg piece of dry tissue was homogenized in 6 ml of Chloroform-Methanol (2:1 v:v); lipids were quantified according to the method of Barnes & Blackstock (1973), using cholesterol as standard (Sigma-Aldrich, St. Louis, MO, USA).

### Statistical Analyses

The differences in (i) polyp expansion and (ii) protein, carbohydrate, lipid concentrations and OM composition of *A. acaule* among several seasons were assessed across sampling times by multivariate analyses.

The design consisted of two factors: Season (S, as fixed factor with four levels) and Sampling Time (T(S)), as random factor with three levels, nested in Season), with *n* = 3 for each combination of factors. Multivariate and univariate permutational analysis of variance (PERMANOVA, McArdle & Anderson, 2001) was based on Euclidean distances of untransformed data of polyp expansion and previously normalized data of proteins, carbohydrates, lipids and OM composition, using 9,999 random permutations of the appropriate units (Anderson & Braak, 2003). When significant differences were encountered (*p* < 0.05), post-hoc pairwise tests for the fixed factor were carried out, to ascertain the consistency of the differences among seasons. Because of the restricted number of unique permutations in the pairwise tests, *p* values were obtained from Monte Carlo samplings. For illustrating differences in the composition of OM, significant terms were plotted using canonical analysis of principal coordinates (CAP) (Anderson & Willis, 2003) for the factor Season. To determine whether the environmental features (*i.e.*, seawater temperature at −5 m, seawater temperature at −20 m, water transparency) influenced polyp expansion and organic matter composition of *A. acaule*, multivariate multiple regression analyses and distance-based Redundancy Analyses (dbRDA, Stepwise regression) were performed (DistLM, McArdle & Anderson, 2001). The analyses were performed using the software PRIMER v. 6 (Clarke & Gorley, 2006).

## Results

### Environmental variables

**Figure 2:**
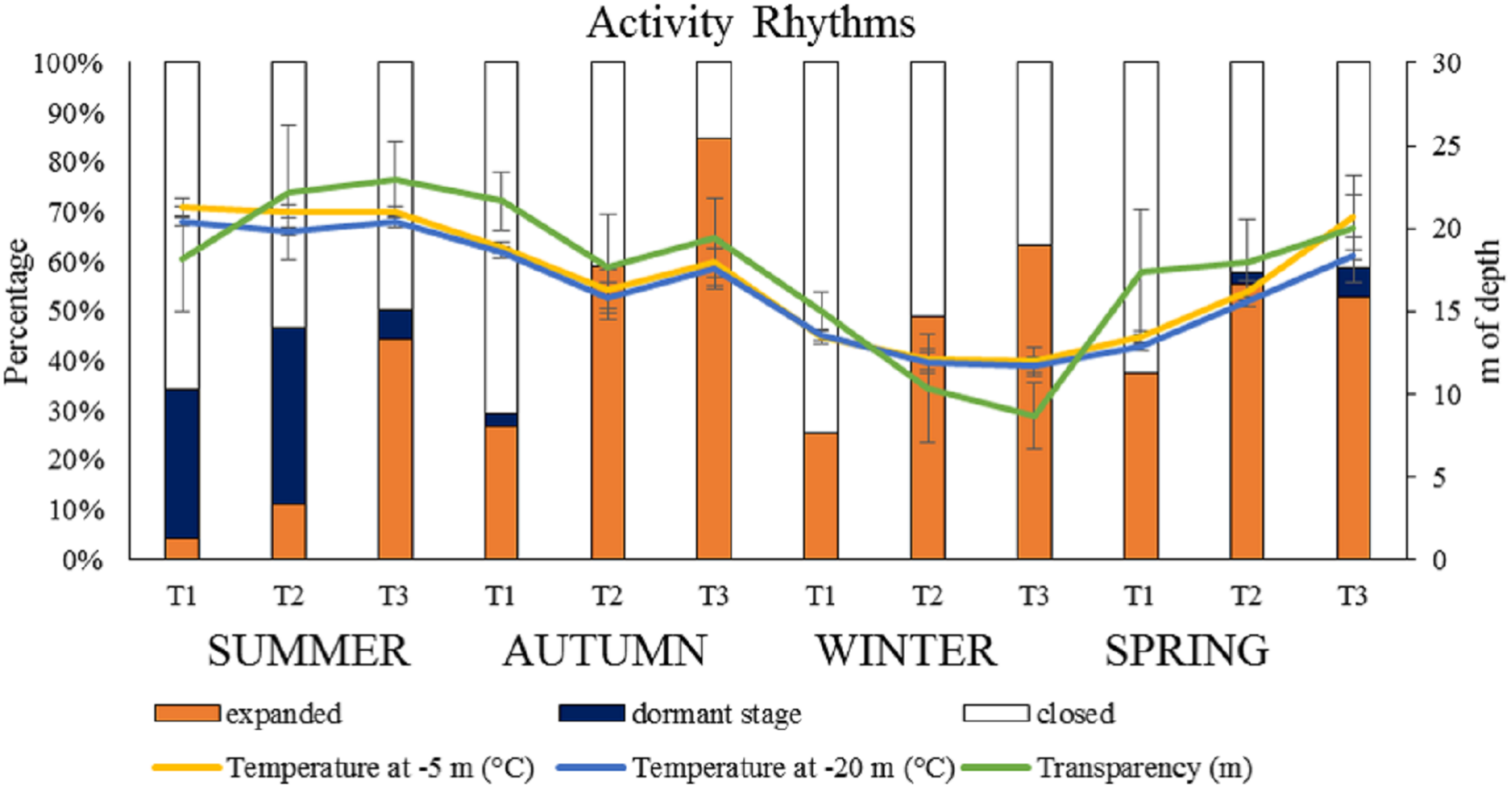
Seasonal polyp expansion of *A. acaule* in relation to the environmental variables.

The tested environmental variables are shown in Fig. 2. The temperatures at −5 m ranged from 11 to 23 °C, whilst at −20 m varied from 11 to 21 °C (Fig. 2). The summer was the warmest season, with a mean of 21 °C at −5 m and 20 °C at −20 m depth, followed by spring (17 and 16 °C, respectively), autumn (16 °C at both depths) and winter (13 and 12 °C, respectively). Water transparency was assessed by Secchi disk which ranged from 5 to 29 m (Fig. 2). It was higher during the summer (approx. 22 m), and decreased in the winter (~11 m, Fig. 2).

### Seasonal polyp expansion

The polyp expansion of *A. acaule* through the entire year is shown in Fig. 2 in relation to the environmental variables investigated. Although associated with a wide variability, a low percentage of polyp expansion occurred in summer together with the phenomenon of dormant stage that was observed in some colonies. In a low number of colonies, dormancy was also present during the late spring and early autumn.

**Figure 3:**
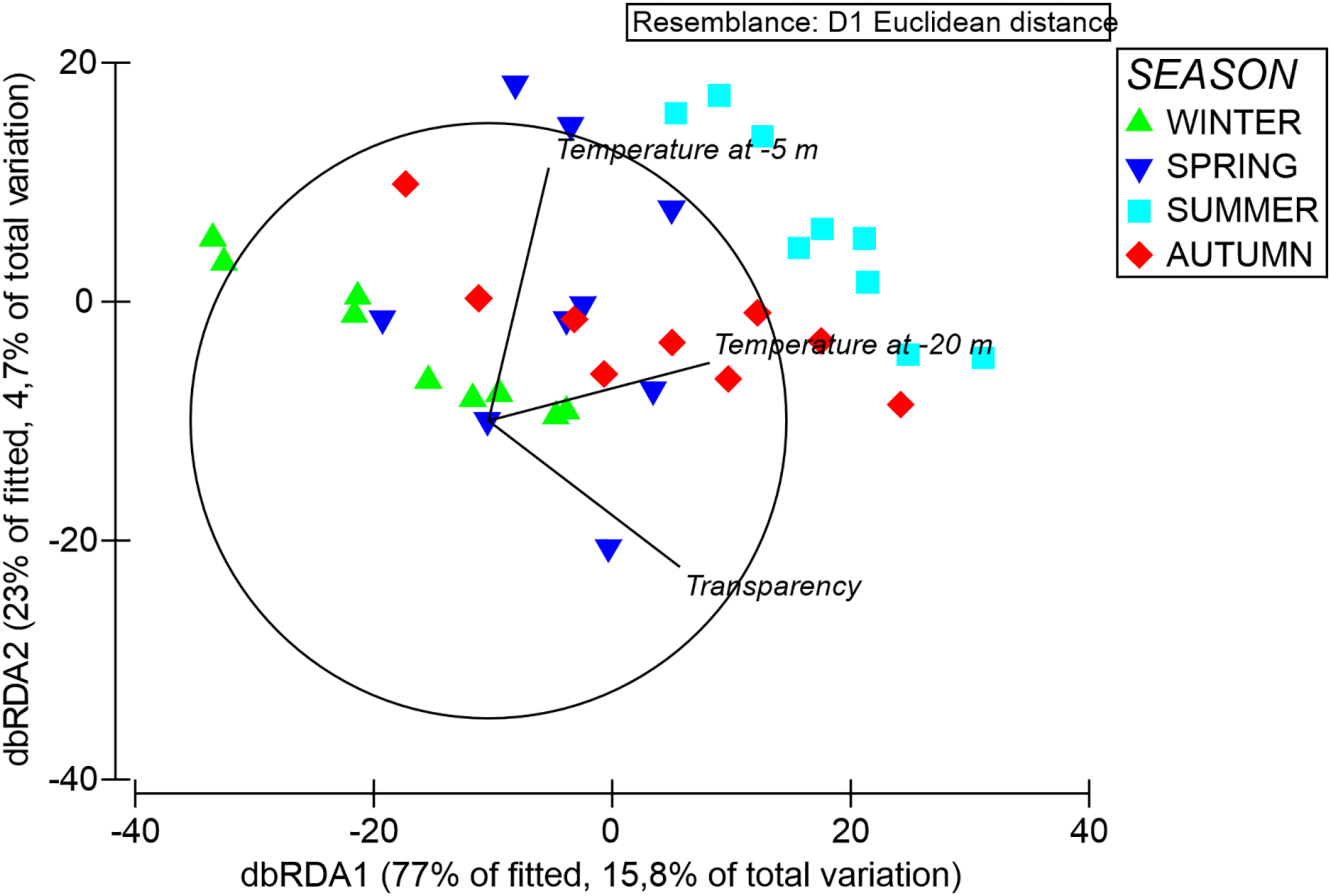
DbRDA ordination after DistLM analysis, showing the relationships among the environmental features and polyp expansion of *A. acaule*.

The PERMANOVA revealed that the polyp expansion of *A. acaule* polyps varied significantly among sampling times within different seasons, while significant seasonal variations had not been observed (Table 1). The results of the DistLM analyses revealed that the temperature at −20 m significantly explained variations in polyp expansion (*p* < 0.05, 14%). In particular, the three considered environmental variables explained cumulatively 20% of polyp expansion variation (Table 2, Fig. 3).

**Table 1:** Results of multivariate permutational analyses of variance (PERMANOVA) in polyp expansion of *A. acaule* among seasons (S) and sampling times (T(S)).

Source	df	MS	Pseudo-F	*P* (perm)
S	3	110.79	1.37	
T(S)	8	80.57	3.96	***
Res	108	20.33		
Total	119			

**Notes:**

df, degree of freedom; MS, mean sum of squares; Pseudo-F, Pseudo-F statistic; *P* (perm), permutational level of probability.

*** *P* < 0.001.

**Table 2:** DistLM analyses estimating the proportion of polyp expansion of *A. acaule* explained singularly (marginal tests) and cumulatively (sequential tests) by environmental features.

Marginal tests			
Variable	*P*	Prop.	
Temperature at −5 m	*	0.12	
Temperature at −20 m	*	0.14	
Transparency	*	0.11	
**Sequential tests**			
**Variable**	***P***	**Prop.**	**Cumul.**
Temperature at −20 m	*	0.14	0.14
Transparency	ns	3.25 E−02	0.17
Temperature at −5 m	ns	3.61 E−02	0.20

**Notes:**

* *p* < 0.05.

ns, not significant; Prop., proportion of explained variance; Cumul., cumulative results.

### Biochemical composition of *A. acaule* organic matter

**Figure 4:**
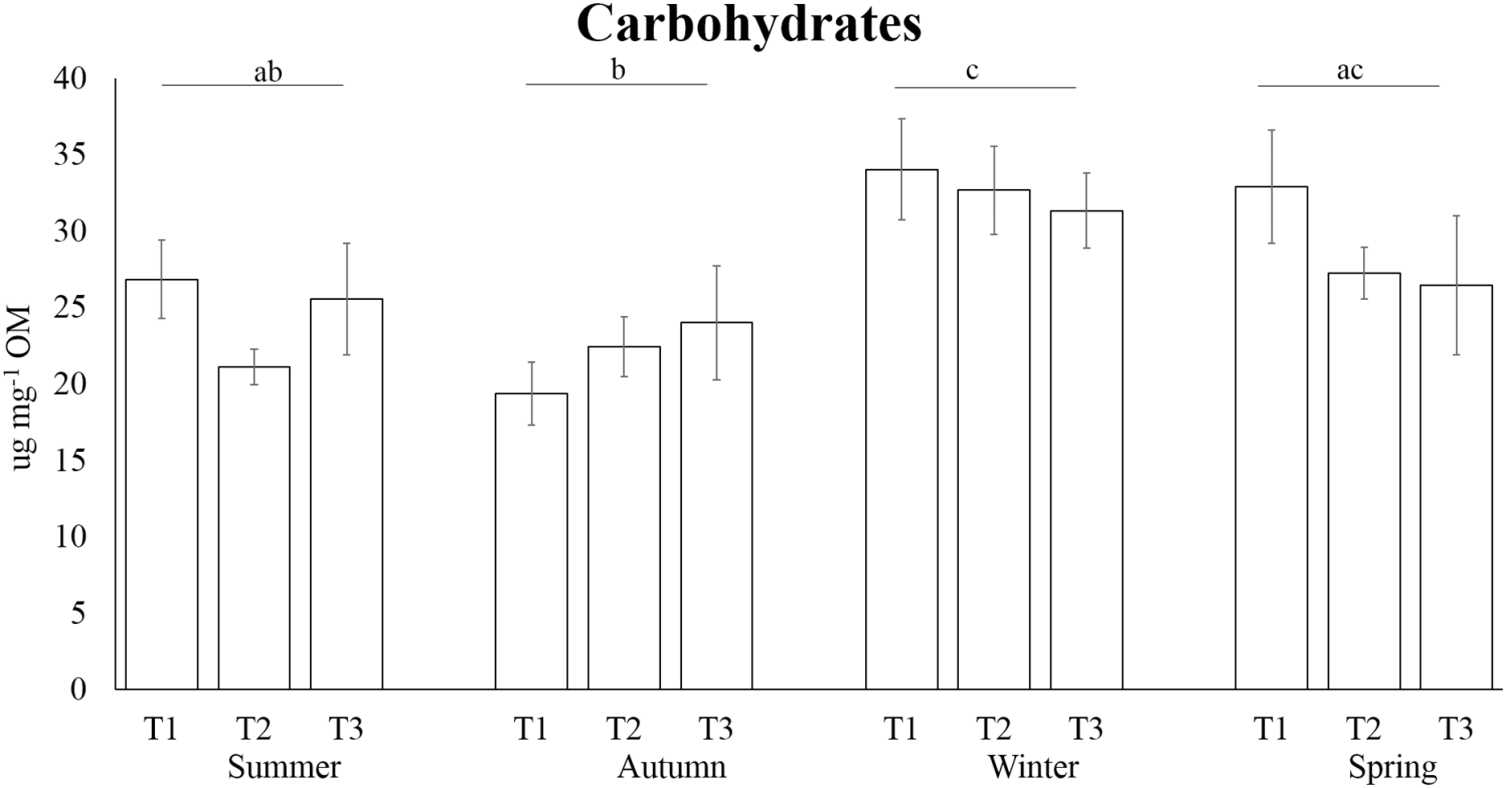
Seasonal carbohydrate concentrations in the tissue of *A. acaule*. Different letters indicate statistically significant differences among seasons (*p* > 0.05).

**Figure 5:**
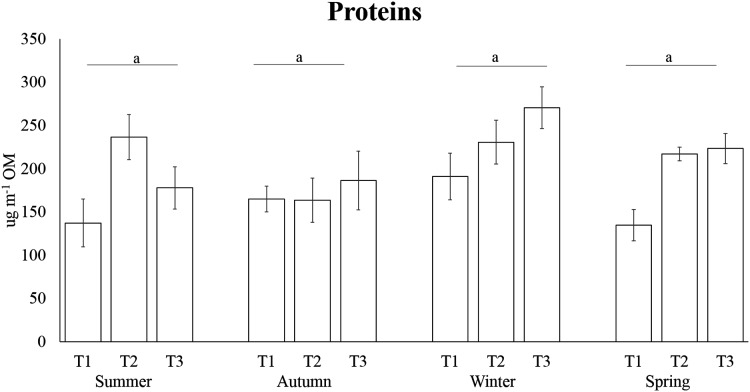
Temporal variation in carbohydrate concentrations of *A. acaule*. Different letters indicate statistically significant differences among seasons (*p* > 0.05).

**Figure 6:**
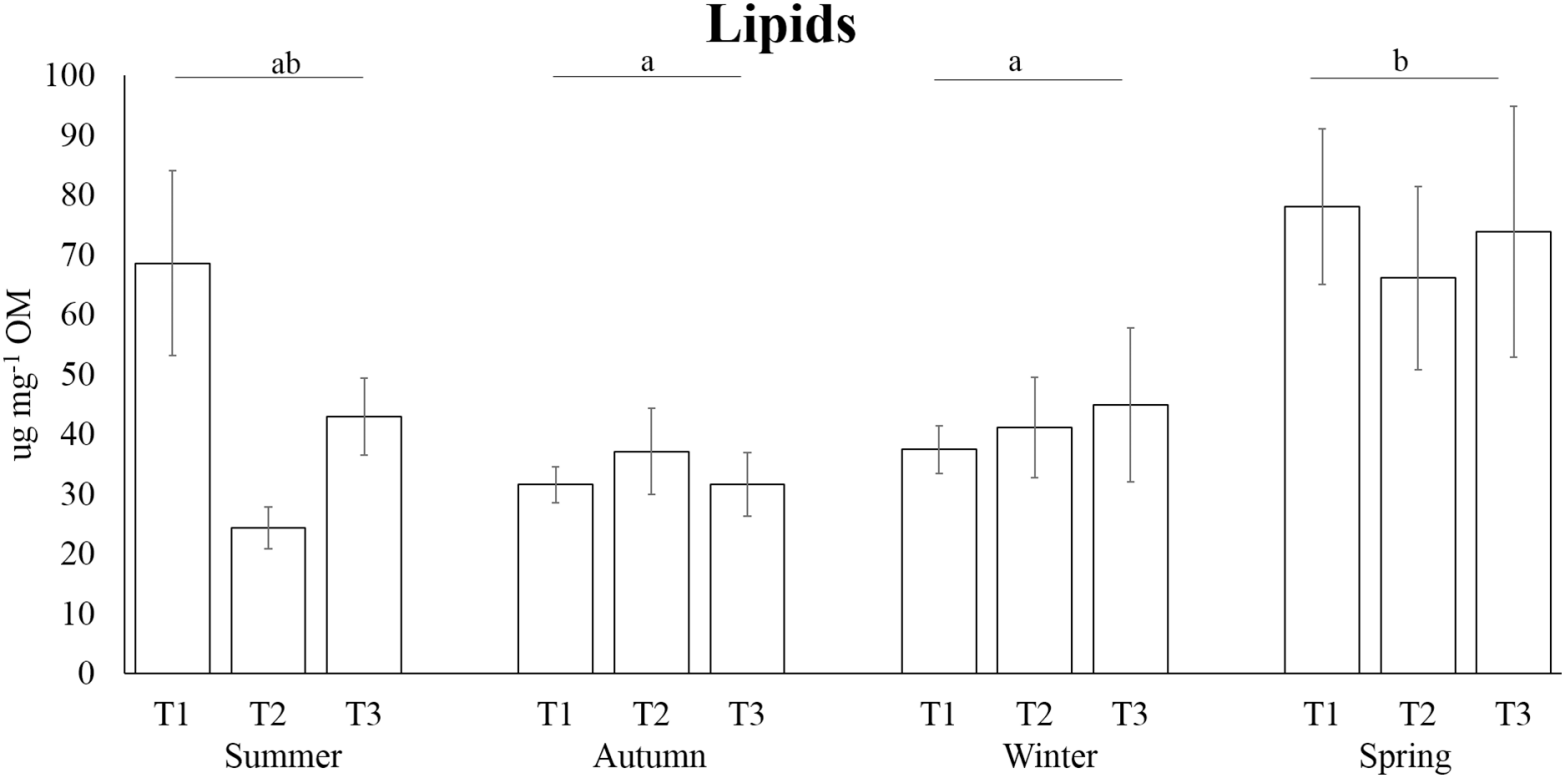
Seasonal lipid concentrations in the tissue of *A. acaule*. Different letters indicate statistically significant differences among seasons (*p* > 0.05).

The highest carbohydrate contents were found in winter (32.69 ± 1.50 µg mg^−1^ OM), followed by the concentrations detected in spring (28.89 ± 2.03 µg mg^−1^ OM) and summer (24.52 ± 1.59 µg mg^−1^ OM); the lowest concentrations were reported in autumn with an average of 21.94 ± 1.51 µg mg^−1^ OM (Fig. 4). Carbohydrate concentration in winter was significantly higher than autumn and winter, while in autumn it was significantly lower than winter and spring (Table 3). PERMANOVA results revealed that protein levels varied significantly across sampling times of several seasons, while no differences have been observed among seasons (Table 3, Fig. 5). The highest lipid contents were found in spring, followed by the concentrations detected in summer and winter; the lowest concentrations were reported for autumn (Fig. 6). Differences in lipid contents did not vary among sampling times, while significant seasonal differences were found (Table 3). In particular, the lipid concentrations in spring were significantly higher than autumn and winter.

**Table 3:** Results of permutational analyses of variance (PERMANOVA) in carbohydrate, protein and lipid concentrations of *A. acaule* among seasons (S) and sampling times (T(S)).

		Carbohydrates			Proteins			Lipids		
Source	df	MS	Pseudo-F	*P* (perm)	MS	Pseudo-F	*P* (perm)	MS	Pseudo-F	*P* (perm)
S	3	5.02	9.40	**	2.10	1.17		4.58	6.32	*
T(S)	8	0.53	0.82	ns	1.79	2.98	*	0.72	1.13	ns
Res	24	0.65			0.60			0.64		
Total	35									

**Notes:**

df, degree of freedom; MS, mean sum of squares; Pseudo-F, Pseudo-F statistic; *P* (perm), permutational level of probability; ns, not significant.

* *P* < 0.05.

** *P* < 0.01.

**Figure 7:**
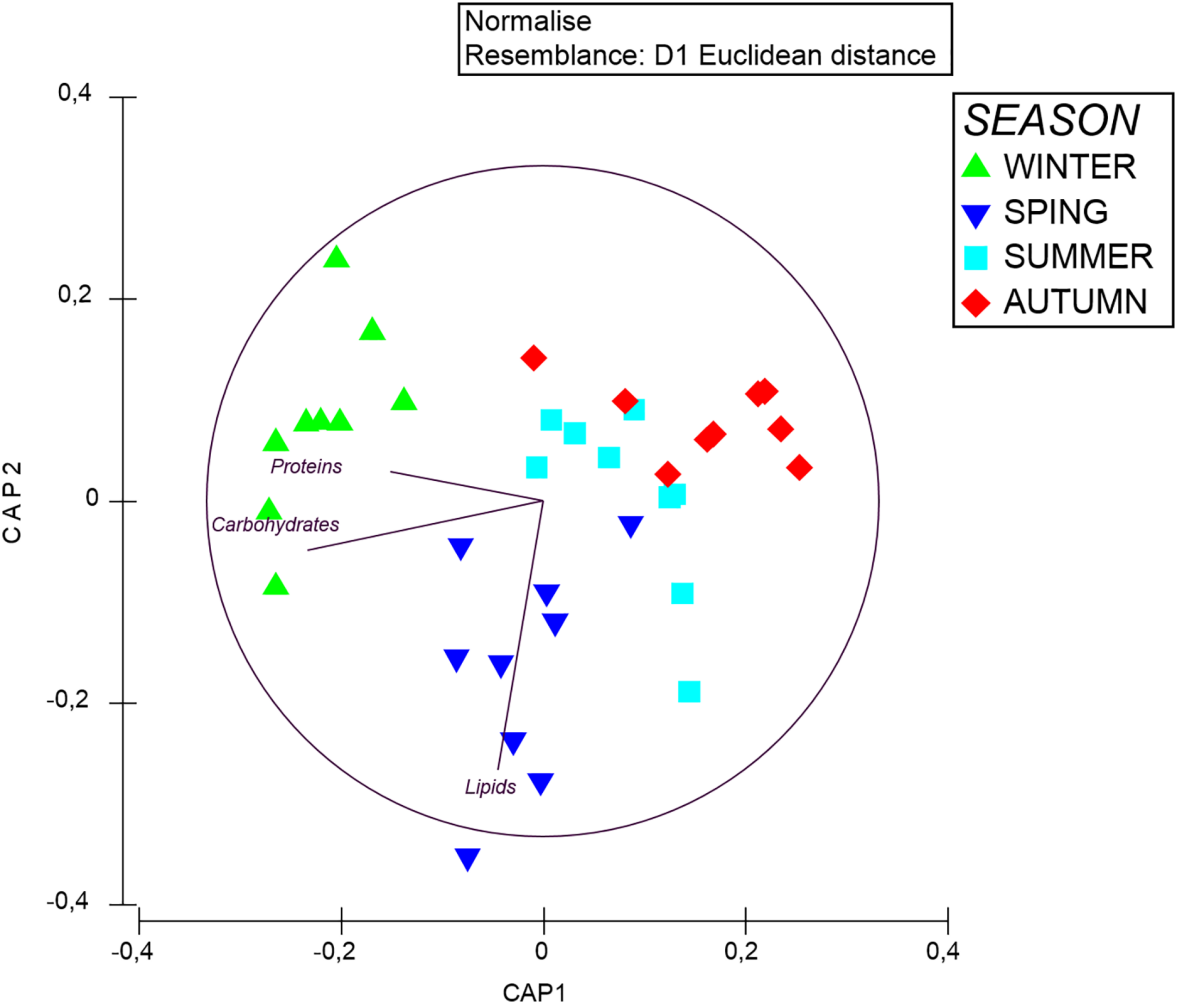
CAP plot of organic matter composition in tissue of *A. acaule* in different seasons. Vectors represent the correlations of each concentrations of carbohydrates, proteins and lipids.

The multivariate PERMANOVA tests revealed that the biochemical composition varied significantly among seasons (Table 4). The pairwise comparisons showed significant differences in OM composition between winter and autumn and between autumn and spring. The CAP plot showed a segregation between seasons (Fig. 7), which is mostly explained by the increased contents of proteins and carbohydrates during the winter and an increment of lipids during the spring.

**Table 4:** Results of multivariate permutational analyses of variance (PERMANOVA) in organic matter composition of *A. acaule* among seasons (S) and sampling times (T(S)).

Source	df	MS	Pseudo-F	*P* (perm)	
S	3	11.70	3.84	**	
T(S)	8	3.05	1.61	ns	
Res	24	1.90			
Total	35				
**Groups**	**t**	***P* (MC)**	**Groups**	**t**	***P* (MC)**
WI *vs* SP	2.05	ns	SP vs SU	1.30	ns
WI *vs* SU	1.69	ns	SP vs AU	2.88	*
WI *vs* AU	3.86	**	SU vs AU	0.80	ns

**Notes:**

df, degree of freedom; MS, mean sum of squares; Pseudo-F, Pseudo-F statistic; *P* (perm), permutational level of probability; *P* (MC), probability level after Monte Carlo simulations; t, pairwise tests; ns, not significant; WI, winter, SP, spring, SU, summer and AU, autumn.

* *P* < 0.05.

** *P* < 0.01.

**Figure 8:**
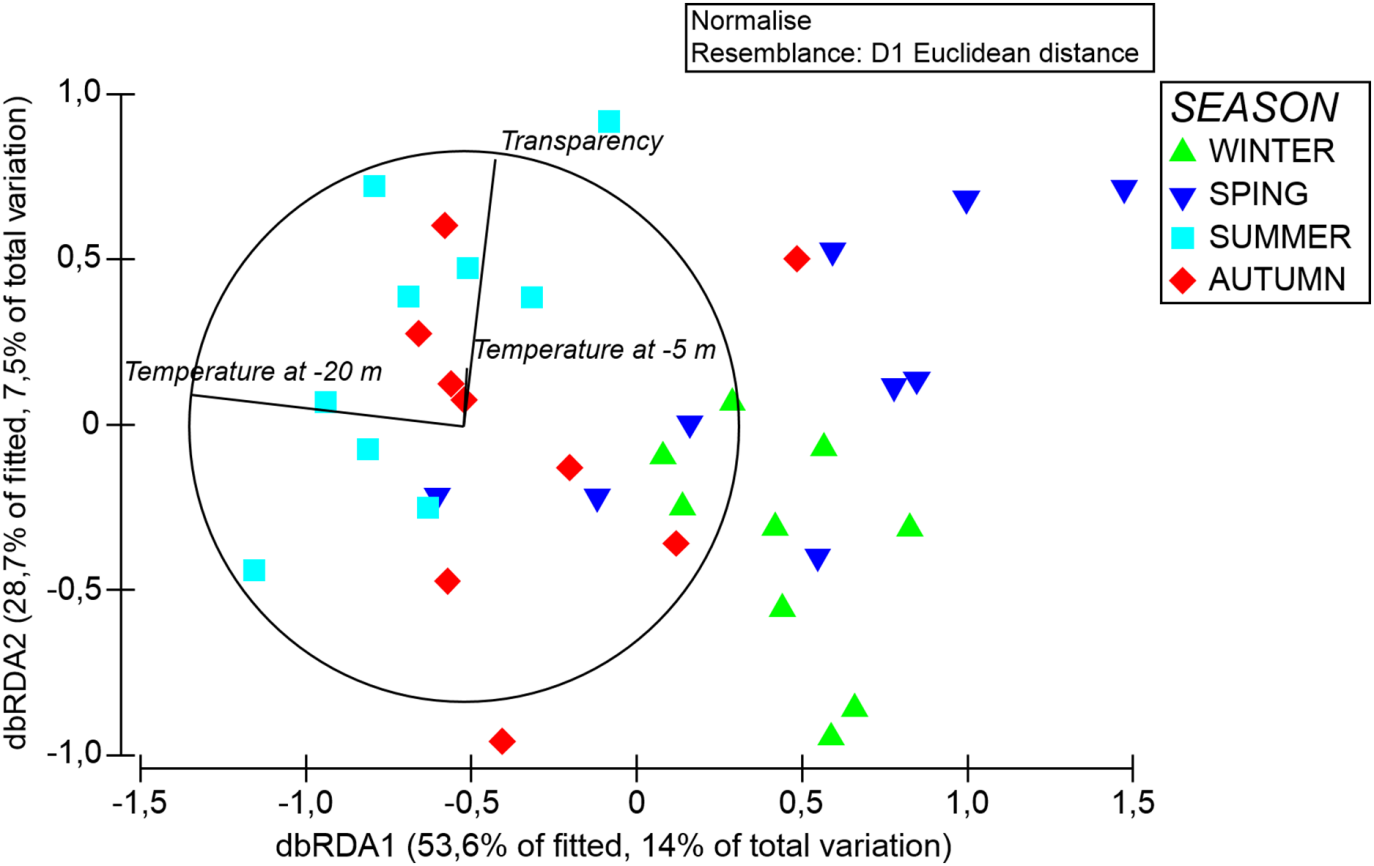
DbRDA ordination after DistLM analysis, showing the relationships among the environmental features and organic matter composition of *A. acaule*.

The results of the DistLM analyses revealed that the temperature at −20 m explained variations in organic matter composition significantly (*p* < 0.05, 9.35%), followed by temperature at −5 m and water transparency, which altogether explained cumulatively 26% of organic matter composition variation (Table 5). This was shown by the dbRDA analysis where these environmental variables cumulatively explained about 21% of organic matter composition variation along the first two axes (Fig. 8).

**Table 5:** DistLM analyses estimating the proportion of organic matter composition in *A. acaule* explained singularly (marginal tests) and cumulatively (sequential tests) by environmental features.

Marginal tests			
Variable	*P*	Prop.	
Temperature at −5 m	ns	7.85E−02	
Temperature at −20 m	*	9.35E−02	
Transparency	ns	7.58E−02	
**Sequential tests**			
**Variable**	***P***	**Prop.**	**Cumul.**
Temperature at −20 m	*	9.35E−02	9.35E−02
Temperature at −5 m	*	0.1	0.20
Transparency	ns	6.58E−02	0.26

**Notes:**

* *p* < 0.05.

ns, not significant; Prop., proportion of explained variance; Cumul., cumulative results.

## Discussion

The findings of this study couples seasonal polyp activity and nutritional condition with key environmental fluctuations in a slow-growing, passive suspension feeder in warm temperate waters. Polyp activity in the field highlighted a high variability that was loosely dependent on different seasons, suggesting an inter-individual variability in the response to water column changes (Duchêne et al., 2000; Duchêne, 2017). The complex dynamics of polyp expansion and the trophic status of *A. acaule* have revealed that, among the environmental variables investigated, water temperature greatly influenced polyp activity and organic matter composition of the octocoral.

Similar to previous studies on anthozoan polyp activity (Garrabou, 1999; Rossi, 2002; Previati et al., 2010; Rossi, Rizzo & Duchêne, 2019), the current study found a significant negative relationship between temperature and polyp activity. Previati et al. (2010) showed that respiration is the main factor conditioning polyp contraction at high temperatures, a result previously suggested by Coma et al. (2002). The high variability detected in the polyp expansion can be explained by the sensitivity of polyps to water temperature, and especially seston abundance and quality (Rossi, Rizzo & Duchêne, 2019).

Seston availability affects Mediterranean gorgonians (Cnidaria, Octocorallia) differently according to species-specific trophic strategies (*i.e.*, mixotrophic and heterotrophic) and population density (Rossi & Rizzo, 2021). Likewise, complex hydrodynamic conditions, including water temperatures varying in time and space, and the water current regime, act simultaneously to shape polyp activity (Rossi, Rizzo & Duchêne, 2019). Water movement may increase plankton concentrations (Sebens & DeRiemer, 1977; Palardy, Grottoli & Matthews, 2006; Rossi & Rizzo, 2021), affecting the polyp expansion. The cumulative effect of seston availability thus appears to be a major factor controlling spawning behaviour as described below.

In the present study, we observed significant differences in carbohydrate and lipid concentrations among seasons, while proteins exhibited great variability across sampling times. Protein levels seem to be lower than values found in *Paramuricea clavata* (Rossi et al., 2006) and *Leptogorgia sarmentosa* (Rossi, 2002); it may be due to the proteinic skeleton of these gorgonians. However, the lipid and carbohydrate concentrations have been suggested as a proxy of resource mobilization (Clarke, 1977; Costello, 1998), and along with Heat Shock Protein (HSP) expression, can be used as biomarkers of environmental changes and food availability for benthic suspension feeders (Rossi, Snyder & Gili, 2006). Our results show carbohydrate concentrations in line with the data reported in the literature for other anthozoans (Ben-David-Zaslow & Benayahu, 1999; Rossi, 2002; Rossi et al., 2006; Rossi & Tsounis, 2007). Lipid concentrations were lower than values found in *Corallium rubrum* by Rossi & Tsounis (2007) and in *Eunicella singularis* (Gori et al., 2007). The highest lipid concentrations in *A. acaule* were found during the spring. Before summer aestivation, organisms reproduce and need a significant quantity of energy to compensate for the gonadal release and the reduced food availability (Coma et al., 2000; Rossi et al., 2006). Indeed, *A. acaule* is gonochoristic and a surface brooder that retains eggs on the external surface of female colonies with mucous strings (Garrabou, 1999; Fiorillo et al., 2013; Teixidó et al., 2016), spawning once a year in late spring-early summer (Fiorillo et al., 2013) in coincidence with its higher lipid contents. Rapidly increasing solar irradiance during the spring has been suggested as the trigger for oocyte maturation and vitellogenesis, nevertheless other underlying mechanisms could be playing a key role (Fiorillo et al., 2013). Summer is characterized by transparent and warm waters, with low plankton and seston concentration (Rossi & Gili, 2005). These conditions impose high basal metabolic energy consumption on benthic suspension feeders (Coma et al., 2000; Rossi et al., 2006; Previati et al., 2010). After the summer, the stratified water column breakdowns as a consequence of autumn storms, mixing the available food and influencing activities and behaviours of benthic organisms in the complex coastal hydrodynamics (Estrada, 1996; Rossi & Gili, 2005; Rossi et al., 2019a). After spawning, the energy invested (Rossi et al., 2006; Rossi & Tsounis, 2007; Viladrich et al., 2017) can be partially recovered in the autumnal peak of primary production (Estrada, 1996; Rossi & Gili, 2005; Rossi et al., 2019b). However, in late autumn, benthic suspension feeders face a period of food scarcity due to the high resuspension of low-quality organic matter (Rossi et al., 2019b). Carbohydrates and lipids reach minimum levels in autumn in many octocorals (Rossi, 2002; Rossi et al., 2006; Rossi & Tsounis, 2007; Gori et al., 2012). This feeding stress is also observed in the HSP 70 and 90 expression, which is especially related to carbohydrate mobilization (Rossi, Snyder & Gili, 2006).

The aestivation process may lead to dormancy states (Coma et al., 2000). We could observe the aestivation phenomenon in *A. acaule* during the summer and, to a minor extent, in the late spring and early autumn. Among benthic fauna, periods of activity that alternate to dormancy are common (Boero & Fresi, 1986; Boero et al., 1986; Turon, 1992; Turon & Becerro, 1992). Contrary to what is observed in warm temperate water, seasonality in the dynamics of marine benthic suspension feeders is characterized by winter dormancy in cold temperate seas (Coma et al., 2000). Generally, the Mediterranean aestivation is characterized by a summer energy shortage (reduced polyp activity, colony dormancy, low oxygen consumption, depletion of energy storage) and a decrease of energy investment in secondary production (*i.e.*, growth and reproduction) during the summer period, however the aestivation processes can differ widely among taxa (Coma et al., 2000; Coma et al., 2002).

Studies on octocorals showed that at high temperatures the metabolic rate decreases in a dormancy-like stage (Coma et al., 2000; Previati et al., 2010). The increase in temperature reflects high respiration rates, reducing the available energy storage of benthic suspension feeders (Rossi et al., 2006). Anthozoans, indeed, are characterized by a decrease in investment in secondary production and in feeding activity during the summer period (Coma et al., 1998), with some species exhibiting non-feeding periods (Garrabou, 1999). The role of resting stages has been recognized as a key factor in life cycle dynamics and in the shaping of marine assemblages in some studies that focus on temporal fluctuations, not only in benthic invertebrates but also in phyto-and zooplankton communities (Boero et al., 1996; Marcus & Boero, 1998; Montresor et al., 2013).

The importance of discerning between seasonal energy invested or stored during periods of insufficient food (or energy and nutrient) availability has been discussed (Clarke, 1977; Epp, Bricelj & Malouf, 1988; Alonzo, Mayzaud & Razouls, 2000). Metabolic adjustments aimed to reduce energy losses could be decisive for avoiding mass mortality events of benthic suspension feeders that have been reported in the summer (Cerrano et al., 2000; Garrabou et al., 2009). Understanding the capability to store energy, coupled with benthic suspension feeding activity, could be key factors to help understand the mass mortalities detected in the Mediterranean Sea. It has been suggested that feeding capabilities will be constrained by temperature shifts in the near future (Galli et al., 2016) under the scenario of warm temperate seas, combined with water stratification in shallow areas and a depletion of primary productivity (Galli, Solidoro & Lovato, 2017).

## Conclusions

Our findings demonstrate a food shortage in late summer and late autumn for the benthic suspension feeder *A. acaule*. Polyp activity highlighted a high variability that was only slightly dependent on seasons, while the aestivation phenomenon, a resting period in which the Anthozoan is not capable of any polyp activity, during the warm months has been quantified. This research supports the hypothesis that some Mediterranean benthic suspension feeders exhibit a seasonal pattern of trophic status, characterized by aestivation. Further research will elucidate the complex alterations driven by climate change in marine animal forests and in their processes of carbon immobilization (Coppari, Zanella & Rossi, 2019; Rossi & Rizzo, 2020), as food quantity and quality will be central to face fast environmental changes and anthropogenic stressors (Rossi, Rizzo & Duchêne, 2019). The knowledge of energy fluxes in these benthic organisms related to changes in environmental variables is important to promote conservation measures addressed to the mitigation of human-induced and climate-driven changes.

## Supplemental Information

10.7717/peerj.12032/supp-1 Supplemental Information 1 Raw Data.

## Additional Information and Declarations

### Data Availability

The following information was supplied regarding data availability: The raw measurements are available in the Supplemental File.
